# Quantile graphs for EEG-based diagnosis of Alzheimer’s disease

**DOI:** 10.1371/journal.pone.0231169

**Published:** 2020-06-05

**Authors:** Aruane M. Pineda, Fernando M. Ramos, Luiz Eduardo Betting, Andriana S. L. O. Campanharo

**Affiliations:** 1 Department of Biostatistics, Institute of Biosciences, São Paulo State University (UNESP), Botucatu, São Paulo, Brazil; 2 National Institute for Space Research (INPE), Earth System Science Center (CCST), São José dos Campos, São Paulo, Brazil; 3 Department of Neurology, Psychology and Psychiatry, Institute of Biosciences, Botucatu Medical School, São Paulo State University (UNESP), Botucatu, São Paulo, Brazil; 4 Department of Biostatistics, Institute of Biosciences, São Paulo State University (UNESP), Botucatu, São Paulo, Brazil; University of Craiova, ROMANIA

## Abstract

Known as a degenerative and progressive dementia, Alzheimer’s disease (AD) affects about 25 million elderly people around the world. This illness results in a decrease in the productivity of people and places limits on their daily lives. Electroencephalography (EEG), in which the electrical brain activity is recorded in the form of time series and analyzed using signal processing techniques, is a well-known neurophysiological AD biomarker. EEG is noninvasive, low-cost, has a high temporal resolution, and provides valuable information about brain dynamics in AD. Here, we present an original approach based on the use of quantile graphs (QGs) for classifying EEG data. QGs map frequency, amplitude, and correlation characteristics of a time series (such as the EEG data of an AD patient) into the topological features of a network. The five topological network metrics used here—clustering coefficient, mean jump length, betweenness centrality, modularity, and Laplacian Estrada index—showed that the QG model can distinguish healthy subjects from AD patients, with open or closed eyes. The QG method also indicates which channels (corresponding to 19 different locations on the patients’ scalp) provide the best discriminating power. Furthermore, the joint analysis of delta, theta, alpha, and beta wave results indicate that all AD patients under study display clear symptoms of the disease and may have it in its late stage, a diagnosis known a priori and supported by our study. Results presented here attest to the usefulness of the QG method in analyzing complex, nonlinear signals such as those generated from AD patients by EEGs.

## 1 Introduction

Alzheimer’s disease (AD) is the main cause of dementia in people over 65 years of age, affecting nearly 25 million people throughout the world [1]. AD is marked primarily by progressive cognitive impairment, loss of memory, and disorientation of time and space. [2]. With an unknown cause, AD usually evolves slowly, following a specific pathway that first involves the hippocampus, then spreads out to association areas in parietal, lateral temporal, and frontal regions, eventually affecting all regions of cortex [3, 4]. AD is irreversible. Thus, the earlier a diagnosis can be made, the sooner the treatment can be started, with a higher chance of success in slowing down the progression of the disease.

Currently, definitive diagnosis of AD is made on the examination of the brain tissue accessed by biopsy or necropsy [5]. Since it is possible to be sure that he or she had AD only after the patient’s death, clinical diagnosis is made by excluding other causes of dementia. However, in recent years there has been considerable research toward the diagnosis of AD using biological markers (biomarkers); see [6–9] for a review. Classified into four basic groups, biochemistry, genetics, neurophysiology, and neuroimaging, AD biomarkers should carry enough information on pathophysiologic processes active in AD so as to allow the detection of the disease’s precursor clues before the symptoms onset [7].

Electroencephalography (EEG), in which the electrical brain activity is recorded in the form of time series and analyzed using signal processing techniques, is a well-known neurophysiological AD biomarker. EEG is noninvasive, low-cost, has a high temporal resolution, and provides valuable information about brain dynamics in AD [10–15]. Recently, computer-aided classification methods have been developed and applied to EEG signals to distinguish among patients with AD, healthy controls, and patients with mild cognitive impairment [9]. AD affects the characteristics of EEGs. Consequently, EEG analysis can provide useful information about the dynamics of the brain due to AD. Slowing EEG, decreased EEG coherence, and decreased EEG complexity are the most distinctive traits in the EEG caused by AD [16]. Nonetheless, the diagnosis of AD from EEG data is still open research topic, and it comes without surprise the wealth of methods proposed in the medical and related literature. These methods, based on Fast Fourier Transform (FFT) [17–19], Wavelet Transform (WT) [20–23], Phase-Space Reconstruction [24–26], Eigenvector Methods (EMs) [27, 28], Time Frequency Distributions (TFDs) [29], and the Auto-Regressive Method (ARM) [30], generally require from the input signal one or more of the following assumptions: stationarity, high time or frequency resolution, and/or a high signal-to-noise ratio.

In [31], we introduced a simple method for transforming a time series into graphs or networks, called the quantile graph (QG) method. The QG method maps relevant properties of the original time series, such as periodicity or randomness, into the topological features of the resulting graph. The QG method has been applied to quantify the Hurst exponent of gaussian white noites and brownian motions [32] and to uncover distinctions between physiological signals of normal individuals and unhealthy patients [13, 31]. We have also used the QG method to distinguish in an EEG the pre-ictal from the ictal stage of an epileptic convulsion [14]. Along the same lines, here we study the QG method as a technique to differentiate patients with AD from control using EEG signals and to indicate which channels (corresponding to 19 different locations on the patients’ scalp) provide the best discriminating power.

After this introduction, Section 2 and 3 present, respectively, the QG method and the network measures used here for the characterization of complex networks. The EGGs used in this research are described in Section 4 while the corresponding results are examined in Section 5. Finally, Section 6 presents the pertinent conclusions.

## 2 Materials and methods

As described in detail previously [14, 31–33], the QG method converts a time series X={x(t)|t∈N,x(t)∈R} into a complex network g={N,A}∈G, with N vertices (or nodes) and A edges (or arcs). In the QG method, each quantile *q*_*i*_ for *i* = 1, 2, …, *Q* of *X* is attributed to a node $n_{i}\in N$ in *g*. Two nodes *n*_*i*_ and *n*_*j*_ are connected with a weighted arc $(n_{i},n_{j},w_{ij}^{k})\in A$ whenever two values *x*(*t*) and *x*(*t* + *k*) belong respectively to quantiles *q*_*i*_ and *q*_*j*_, with *t* = 1, 2, …, *T* and *k* = 1, …, *k*_*max*_ < *T*. Weights $w_{ij}^{k}$ in the weighted directed adjacency matrix, which is denoted as *A*_*k*_, are equal to the number of times *q*_*i*_ at time *t* is followed by *q*_*j*_ at time *t* + *k*. Thus, repeated transitions through the same edge increase the the corresponding weight value [14]. Normalizing *A*_*k*_, it becomes a Markov transition matrix *W*_*k*_, with $\sum_{j}^{Q}w_{ij}^{k}=1$ [32]. As shown in [14, 32], the QG method is weakly dependent on the choice of *Q*. Here, the number of quantiles is given by *Q* ≈ 2*T*^1/3^ [34]. A C-code implementation of our method has been made freely available by Pineda *et al*. [35].

**Fig 1:**
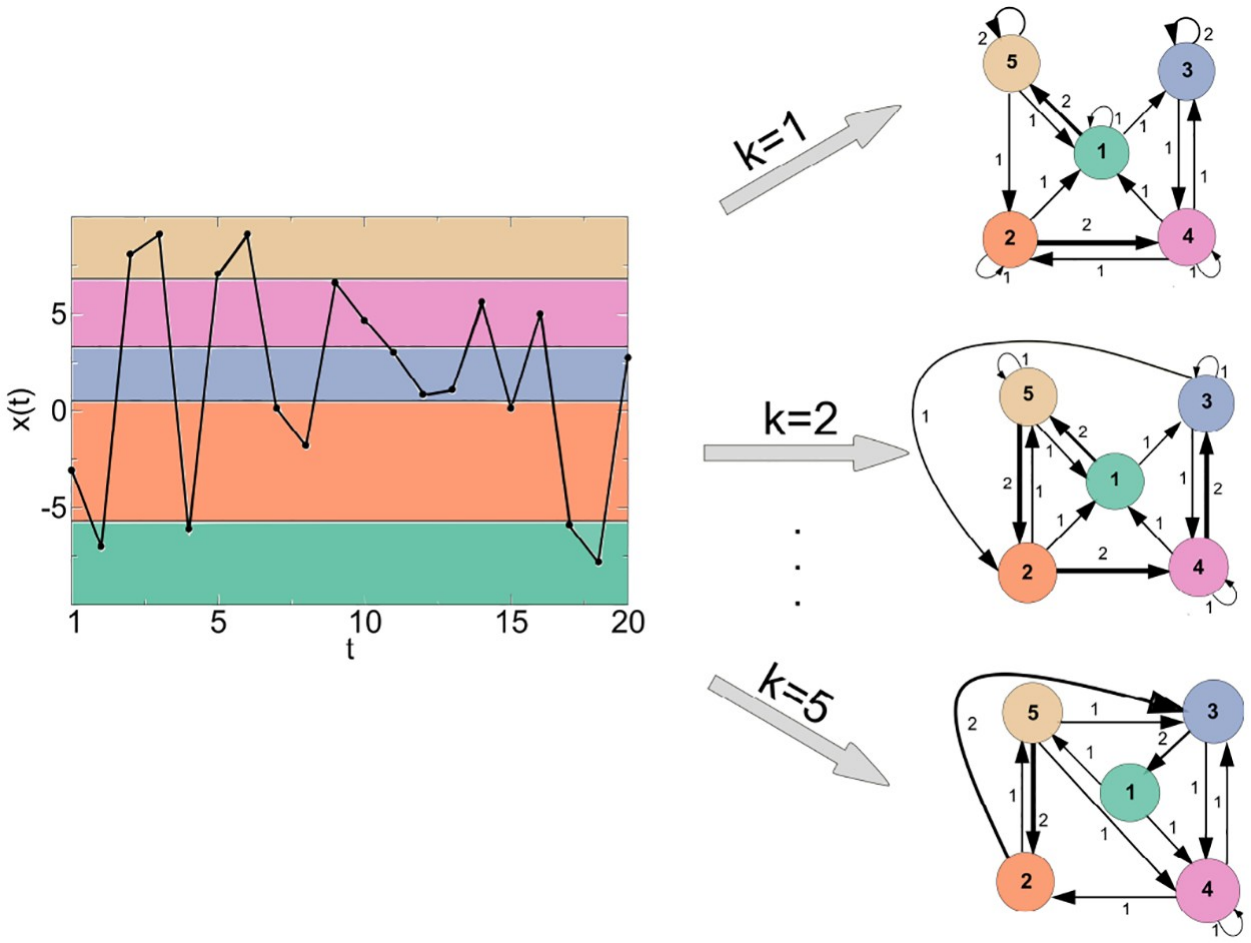
Example of the QG method for a time series with *T* = 20, *Q* = 5, and *k* = 1, 2 and 5. The quantile intervals for the sorted data are given by [*x*(0), *x*(4)[, [*x*(4), *x*(8)[, [*x*(8), *x*(12)[, [*x*(12), *x*(16)[, and [*x*(16), *x*(20)], i.e., [−7.783, −3.050[, [−3.050, 0.829[, [0.829, 4.657[, [4.657, 7.070[, and [7.070, 9.090]. The quantiles are mapped into three networks with N=5 nodes each and arc weights given by (1,1,1), (1,3,1), (1,5,2), (2,1,1), (2,2,1), (2,4,2), (3,3,2), (3,4,1), (4,1,1), (4,2,1), (4,3,1), (4,4,1), (5,1,1), (5,2,1), (5,5,2) for *k* = 1; (1,3,1), (1,5,2), (2,1,1), (2,4,2), (2,5,1), (3,2,1), (3,3,1), (3,4,1), (4,1,1), (4,3,2), (4,4,1), (5,1,1), (5,2,2), (5,5,1) for *k* = 2; and (1,4,1), (1,5,1), (2,3,2), (2,5,1), (3,1,2), (3,4,1), (4,2,1), (4,3,1), (4,4,1), (5,2,2), (5,3,1), (5,4,1) for *k* = 5.

As an illustrative example, Fig 1 shows a simple application of the QG method, *X* with *T* = 20 and *Q* = 5 (colored shading). Quantiles are defined as [*x*(0), *x*(4)[, [*x*(4), *x*(8)[, [*x*(8), *x*(12)[, [*x*(12), *x*(16)[, and [*x*(16), *x*(20)]. For a given *k*, the quantiles are mapped into a network with N=5 nodes and each quantile is assigned to a node $n_{i}\in N$ in the corresponding network *g*. For *k* = 1, 2, and 5, the nodes *n*_*i*_ and *n*_*j*_ are connected with weighted arcs $\left(n_{i},n_{j},w_{ij}^{1}\right)$, $\left(n_{i},n_{j},w_{ij}^{2}\right)$, and $(n_{i},n_{j},w_{ij}^{5})\in A$, respectively. The arc weights are given by (1,1,1), (1,3,1), (1,5,2), (2,1,1), (2,2,1), (2,4,2), (3,3,2), (3,4,1), (4,1,1), (4,2,1), (4,3,1), (4,4,1), (5,1,1), (5,2,1), (5,5,2) for *k* = 1; (1,3,1), (1,5,2), (2,1,1), (2,4,2), (2,5,1), (3,2,1), (3,3,1), (3,4,1), (4,1,1), (4,3,2), (4,4,1), (5,1,1), (5,2,2), (5,5,1) for *k* = 2; and (1,4,1), (1,5,1), (2,3,2), (2,5,1), (3,1,2), (3,4,1), (4,2,1), (4,3,1), (4,4,1), (5,2,2), (5,3,1), (5,4,1) for *k* = 5. Note that the more repeated transitions between the quantiles *q*_*i*_ and *q*_*j*_ occur, the larger the weights between the nodes *n*_*i*_ and *n*_*j*_ are (represented in the corresponding network by thicker lines).

## 3 Network measures

In the recent past, several studies have shown the relevance and usefulness of complex network theory to the comprehension of a wide range of phenomena, across various scientific disciplines, from social sciences to biology [36]. Complex network theory relies on the use of mathematical metrics that can to quantify different features of the network’s topology. Based on the adjacency matrix *A* and Markov transition matrix *W*, we describe the network measures used in this work, namely the clustering coefficient (*CC*), the mean jump length (Δ), the betweenness centrality (*BC*), the modularity (*Mo*), and the Laplacian Estrada index (*LEE*). Code implementations of those measures have been made freely available by Pineda *et al*. [35] and Bounova [37].

### 3.1 Clustering coefficient

Some networks tend to have more links between adjacent vertices, in a way that their topology deviates from that of an uncorrelated random network, in which triangles are sparse. This pattern is called clustering [38], and reflects the segregation of edges into tightly connected neighborhoods. There have been various attempts in the literature to develop a clustering coefficient for weighted networks. Here, the clustering coefficient of a given node *n*_*i*_ is given by [38]:

$$\begin{array}{c} CC_{i}=\frac{1}{s_{i}\left(d_{i}-1\right)}\frac{\sum_{j,d}\left(w_{ij}+w_{id}\right)}{2}\left(a_{ij}a_{jd}a_{id}\right), \end{array}$$ (1)

where *w*_*ij*_ is an element in the weighted matrix *W*, *a*_*ij*_ = 1 if there is a arc from node *n*_*i*_ to node *n*_*j*_, and 0 otherwise. *d*_*i*_ is the total degree of node *n*_*i*_, and *s*_*i*_ is the strength of connectivity of node *n*_*i*_. The global clustering coefficient for the entire network, denoted as *CC*, is defined by the average of the local clustering coefficients over all nodes.

### 3.2 Mean jump length

Given a Markov transition matrix *W* of a graph *g*, it is possible to perform a random walk on it and compute the mean jump length Δ, defined as follows [32]:

$$\begin{array}{c} \Delta =\frac{1}{S}\sum_{s=1}^{}\delta_{s}\left(i,j\right), \end{array}$$ (2)

where *s* = *S* is the total number of jumps, and the length *δ*_*s*_(*i*, *j*) = |*i* − *j*|, with *i*, *j* = 1, …, *Q* being the node indices, as defined by *W*. As described previously [14], a less time-consuming approach for the calculation of Δ, for large *S*, is given by:

$$\begin{array}{c} \Delta =\frac{1}{Q}tr\left(PW^{T}\right), \end{array}$$ (3)

with *W*^*T*^ being the transpose of *W*, *P* a *Q* × *Q* matrix with elements *p*_*i*,*j*_ = |*i* − *j*|, and *tr* the trace operation.

### 3.3 Betweenness centrality

Betweenness is a centrality measure based on shortest paths, widely used in complex network analysis. The betweenness centrality (BC) of a node *n*_*u*_ is given by [39]:

$$\begin{array}{c} BC_{n_{u}}=\sum_{ij}\frac{\sigma (n_{i},n_{u},nj)}{\sigma (n_{i},n_{j})}, \end{array}$$ (4)

where *σ*(*n*_*i*_, *n*_*u*_, *n*_*j*_) is the number of shortest paths between nodes *n*_*i*_ and *n*_*j*_ that go through node *n*_*u*_, *σ*(*n*_*i*_, *n*_*j*_) is the total number of shortest paths between *n*_*i*_ and *n*_*j*_, and the sum is calculated over all pairs *n*_*i*_, *n*_*j*_ of distinct nodes [39, 40]. The betweenness centrality for the entire network, denoted by *BC*, is defined as the average of the local betweenness centralities over all nodes.

### 3.4 Modularity

Recently, the subject of detecting the modular structure of a complex network has gained a large amount of attention [41]. Networks with high modularity present smaller clusters of nodes connected more to each other than to the network at large [42]. Several methodologies have been developed for modules detection and characterization [41]. The goal of a module identification algorithm is to find *P*_*i*_ that maximizes the modularity *M*(*P*_*i*_), where *P*_*i*_ is the set of nodes of module *i*. Given the ensemble P of all partitions, the modularity of P∈P is computed as:

$$\begin{array}{c} M\left(P\right)=\sum_{i=1}^{m}[\frac{e_{i}}{E}-(\frac{d_{i}}{2E}{)}^{2}], \end{array}$$ (5)

with *E* being the total number of edges in the network, *d*_*i*_ the sum of all node degrees in module *i*, and *e*_*i*_ the number of edges within module *i*. In Eq (5), the sum is evaluated over all the *m* modules in the partition *P* [43]. In the present work, we used the algorithm developed by [44] for determining P and calculating *M*(*P*).

### 3.5 Laplacian estrada index

Let *g* be a network without loops and multiple edges. The Laplacian matrix of *g* is the matrix *L* = *D* − *A* where *D* is a diagonal matrix with (*d*_1_, …, *d*_*n*_) on the main diagonal in which *d*_*i*_ is the degree of the node *n*_*i*_. Since *L* is a real symmetric matrix, its eigenvalues *μ*_1_, *μ*_2_, …, *μ*_*n*_ are real numbers. These are referred to as the Laplacian eigenvalues of the underlying network [45]. Let’s assume those to be labelled in a non-increasing manner *μ*_1_ ≥ *μ*_2_ … ≥ *μ*_*n*_. The Laplacian Estrada index of a network *g* is defined as [46]:

$$\begin{array}{c} LEE=LEE\left(g\right)=\sum_{i=1}^{n}e^{\mu_{i}}. \end{array}$$ (6)

## 4 Data

**Fig 2:**
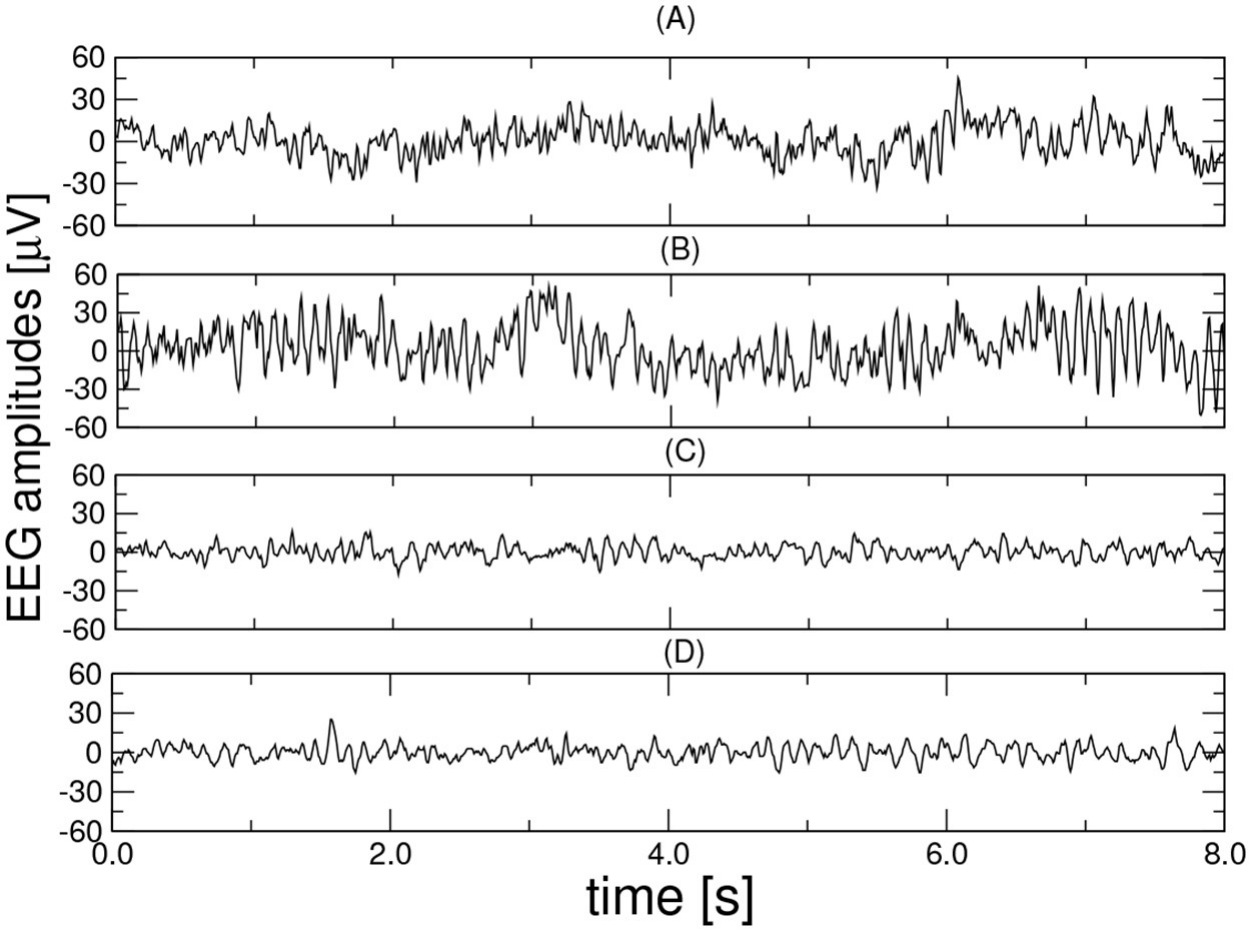
Exemplary EEG segments (channel *F*_7_) from each of the four groups (A, B, C, and D). From top to bottom: health controls, eyes open (group A), health controls, eyes closed (group B), patient with AD, eyes open (group C) and patient with AD, eyes closed (group D).

The database was designed jointly by researchers at Florida State University and it was recorded from the 19 scalp (*F*_*p*1_, *F*_*p*2_, *F*_*z*_, *F*_3_, *F*_4_, *F*_7_, *F*_8_, *C*_*z*_, *C*_3_, *C*_4_, *T*_3_, *T*_4_, *P*_*z*_, *P*_3_, *P*_4_, *T*_5_, *T*_6_, *O*_1_, and *O*_2_) loci of the international 10-20 system using a Biologic Systems Brain Atlas III Plus workstation [47]. The letters F, C, P, O, and T refer to cerebral lobes (F: frontal, C: central, P: parietal, O: occipital, and T: temporal). Recordings, which include four groups (denoted as A, B, C, and D), were made under two rest states: eyes open (groups A and C) by visually fixating and eyes closed (groups B and D) using a linked-mandible reference forehead ground [47]. Groups A and B represent healthy controls and consist of 24 healthy elderly (average age 72, range 61-83), all being negative for any neurological or psychiatric disorders. Groups C and D consist of 24 probable AD patients (average age 69, range 53-85) diagnosed through the National Institute of Neurological and Communicative Disordersand Stroke (NINCDS) and the Alzheimer’s Disease and Related Disorders Association (ADRDA), and Diagnostic and Statistical Manual of Mental Disorders (DSM)-III-R criteria, as described previously [33, 48]. EEG segments of 8 s duration, band-limited to the range of 1-30 Hz, were recorded at a sampling frequency of 128 Hz (free from eye motion and blinking, and myogenic artifacts) and extracted from the EEG recordings. An EEG technician was with each patient during the recordings to monitor the patients’ vigilance state. The database has been provided by Dr. Dennis Duke of Florida State University and made freely available by Campanharo *et al*. [49]. A detailed description of the database can be found in [47]. Exemplary data from channel *F*_7_ are depicted in Fig 2.

## 5 Results

### 5.1 Discriminating between aging and Alzheimer’s disease

**Fig 3:**
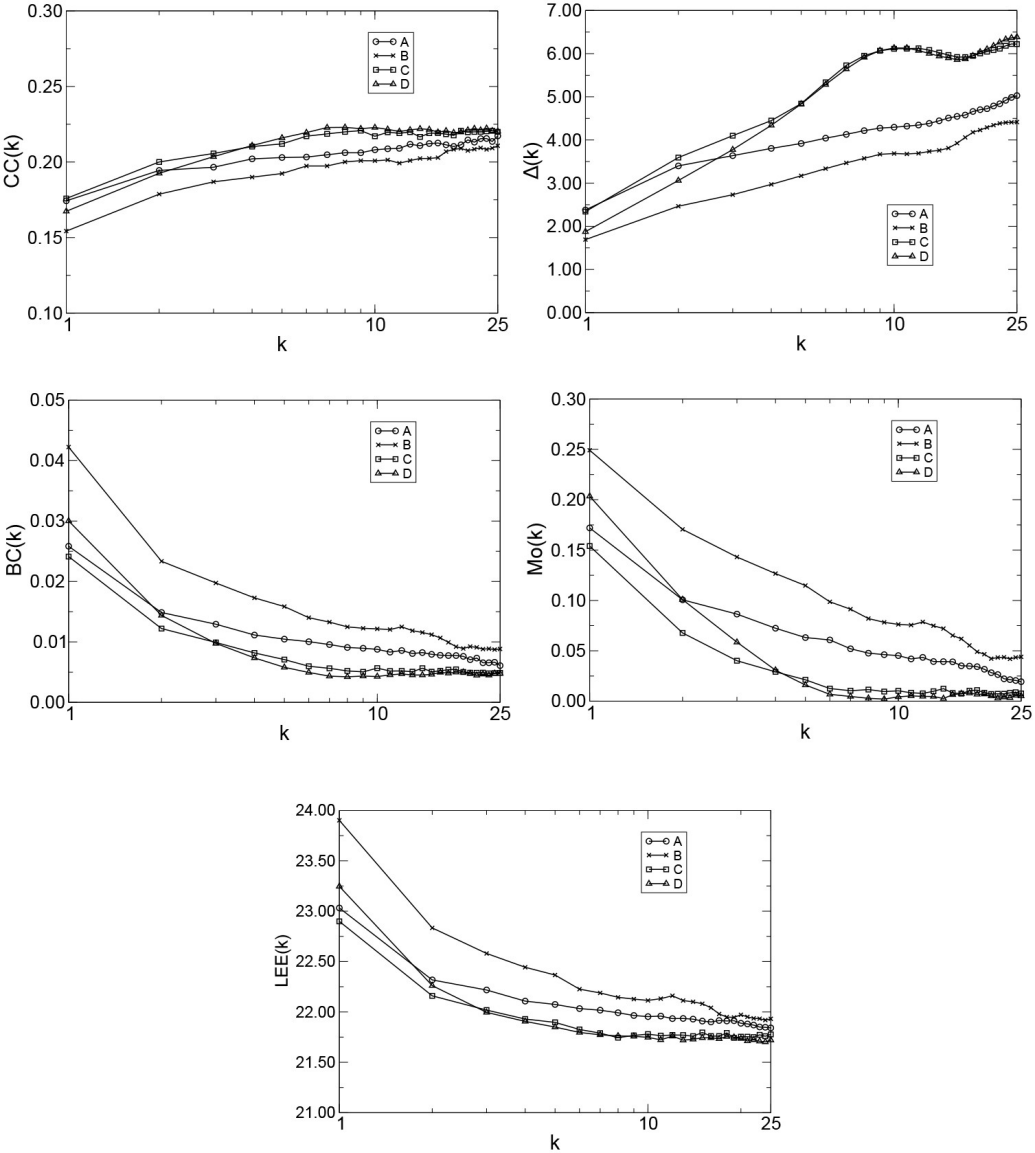
*CC*(*k*), Δ(*k*), *BC*(*k*), *Mo*(*k*), and *LEE*(*k*) versus *k*, *T* = 1, 024, *Q* = 20, and *k* = 1, 2, …, 25 for the groups A (patients from health controls, eyes open), B (patients from health controls, eyes closed), C (patients with AD, eyes open), and D (patients with AD, eyes closed).

We apply the QG method to the problem of discriminating patients with AD from normal controls. The data from channel *F*_7_ was chosen in the simulations due to its closeness to the hippocampal region, which is is one of the first regions of the brain to be affected by AD. As all time series have the same length (*T* = 1, 024), we used *Q* = 2(1, 024)^1/3^ ≈ 20 and *k* = 1, 2, …, 25 in all calculations. Thus, we mapped 2 × 24 × 25 time series into 1,200 quantile graphs (or 1,200 *A*_*k*_ matrices), and therefore, we obtained 1,200 *W*_*k*_ matrices with *Q*^2^ = 400 elements each. Following, for each group and a given *k*, we computed the median over all weighed directed adjacency matrices *A*_*k*_ and obtained the Markov transition matrix of medians. For all groups, we computed *CC*(*k*), Δ(*k*), *BC*(*k*) *Mo*(*k*), and *LEE*(*k*) versus *k* using Eqs (1), (3), (4), (5) and (6), respectively (Fig 3). Note in all cases that the curves for normal controls (A or B) and patients with AD (C or D) form two distinct clusters with maximum separation at approximately *k*_*max*_ = 9 for *CC*(*k*), *k*_*max*_ = 10 for Δ(*k*), *k*_*max*_ = 6 for *BC*(*k*), *k*_*max*_ = 6 for *Mo*(*k*), and *k*_*max*_ = 8 for *LEE*(*k*).

**Fig 4:**
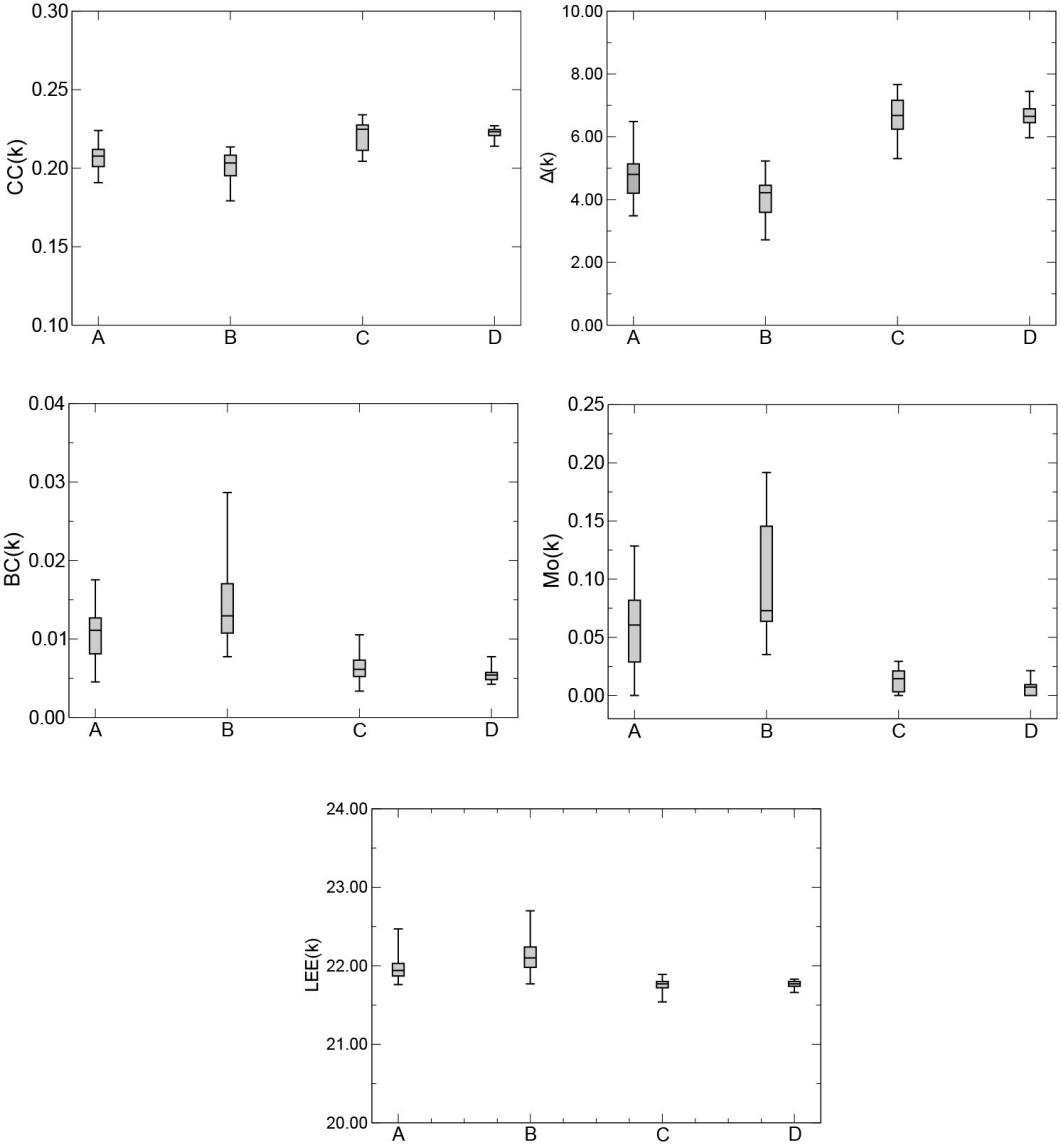
Boxplots of *CC*(*k*), Δ(*k*), *BC*(*k*),*Mo*(*k*), and *LEE*(*k*) for *k*_*max*_ = 9, *k*_*max*_ = 10, *k*_*max*_ = 6, *k*_*max*_ = 6, and *k*_*max*_ = 8, respectively, for the groups A, B, C, and D. Boxplots from patients with different health states show different means (placed at the center of each box), which are 0.2077, 0.2033, 0.2248, and 0.2232 for *CC*(*k*); 4.8010, 4.2190, 6.6790, and 6.6510 for Δ(*k*); 0.0111, 0.0129, 0.0061, and 0.0054 for *BC*(*k*); 0.0605, 0.0728, 0.0145, and 0.0072 for *Mo*(*k*); and 21.9400, 22.1000, 21.7700, and 21.7700 for *LEE*(*k*), respectively.

Fig 4 presents boxplots of *CC*(*k*), Δ(*k*), *BC*(*k*), *Mo*(*k*), and *LEE*(*k*), computed for twelve sample segments each, for the groups A, B, C, and D, and *k*_*max*_ = 9, *k*_*max*_ = 10, *k*_*max*_ = 6, *k*_*max*_ = 6, and *k*_*max*_ = 8, respectively. We observe that, irrespective of the metric used to characterize a given network, the QG method identified health controls with eyes open (group A) and closed (group B), and patients with AD with eyes open (group C) and closed (group D). We performed ANOVA analysis to quantify the sample mean differences found in Fig 4. Table 1 shows a 95% confidence interval and a p-value of less than 0.05 among the sample means of the network measures *CC*, Δ, *BC*, *Mo*, and *LEE* for the groups A, B, C, and D. We also performed a Receiver Operating Characteristic (ROC) analysis [50, 51] in order to quantify how accurately the QG was able to discriminate subjects and/or patients from any two groups under different health conditions. Table 2 shows the areas under the ROC curves (*A*_*ROC*_) of the network metrics *CC*, Δ, *BC*, *Mo*, and *LEE*, for patients in groups A and C, B and D, A and D and B and C. In all cases, the QG method shows an excelent performance in discriminating patients with different health conditions. Comparing the results across the five network metrics, we also observe that Δ provides the best discriminating power.

**Table 1:** Statistical comparison (95% confidence interval, *p* < 0.05) among the sample means of the network measures CC, Δ, BC, Mo, and LEE for the groups A, B, C and, D, through ANOVA.

	*CC*	Δ	*BC*	*Mo*	*LEE*
*CI*_*HU*_	[-0.0221; -0.0071]	[-2.5075; -1.3472]	[0.0012; 0.0078]	[0.0200; 0.0751]	[0.0978; 0.3967]
*p* − *value*	2.3900 x 10^−8^	1.9000 x 10^−14^	2.3360 x 10^−7^	5.5930 x 10^−9^	3.2160 x 10^−7^

*U* = {*C*, *D*}(*unhealthy*)

*H* = {*A*, *B*}(*healthy*)

**Table 2:** Areas under the ROC curves (A_ROC_) of the network measures CC, Δ, BC, Mo, and LEE, for patients in groups A and C, B and D, A and D, and B and C for *k*_*max*_ = 9, 10, 6, 6 and 8, respectively.

	*CC*	Δ	*BC*	*Mo*	*LEE*
$A_{ROC_{AC}}$	0.8472	0.9653	0.8368	0.8785	0.8889
$A_{ROC_{BD}}$	1.0000	1.0000	0.9965	1.0000	0.9583
$A_{ROC_{AD}}$	0.9375	0.9653	0.9167	0.9236	0.9236
$A_{ROC_{BC}}$	0.9167	1.0000	0.9792	1.0000	0.9514

Finally, we used a support vector machine (SVM, [18, 52]), which is a supervised machine for two-class classification problems, to individually classify healthy elderly subjects and patients with AD. Based on the values of *CC*(*k*), Δ(*k*), *BC*(*k*), *Mo*(*k*), and *LEE*(*k*) of 24 healthy subjects (groups A and B) and 24 patients with AD (groups C and D) and the k-fold cross-validation strategy (k = 10), the patients were randomly divided into ten equivalent subsamples. Among the ten subsamples, nine-fold (90% of samples) were considered the training set and the remaining fold (10% of samples) was considered the test set. The values of accuracy (ACC) (100%), sensitivity (SEN) (100%), specificity (SPE) (100%), and area under the curve(AUC) (1.0) show that the QG method is a reliable technique for differentiating patients in different health conditions.

### 5.2 EEG channels influence in the AD detection

To verify the extent to which the electrode location affects the differentiation between normal controls and patients with AD, we used the 19 EEG channels available in our analysis. Analogous to previous case, since for all channels the time series have the same length (*T* = 1, 024), we used *Q* = 2(1, 024)^1/3^ ≈ 20 and *k* = 1, 2, …, 25 in all calculations. Therefore, we mapped 19 × 2 × 24 × 25 time series into 22,800 quantile graphs (or 22,800 *A*_*k*_ matrices), and therefore, we obtained 22,800 *W*_*k*_ matrices with *Q*^2^ = 400 elements each. For all groups and all channels, we calculated *CC*(*k*), Δ(*k*), *BC*(*k*), *Mo*(*k*), and *LEE*(*k*) versus *k*. For a given network measure and channel, *k*_*max*_ was chosen in such way to obtain the maximum separation between the curves of normal controls (groups A or B) and patients with AD (groups C or D) and the average of *A*_*ROC*_, denoted here by ${\hat{A}}_{ROC}$, was computed through the combination between the groups.

**Fig 5:**
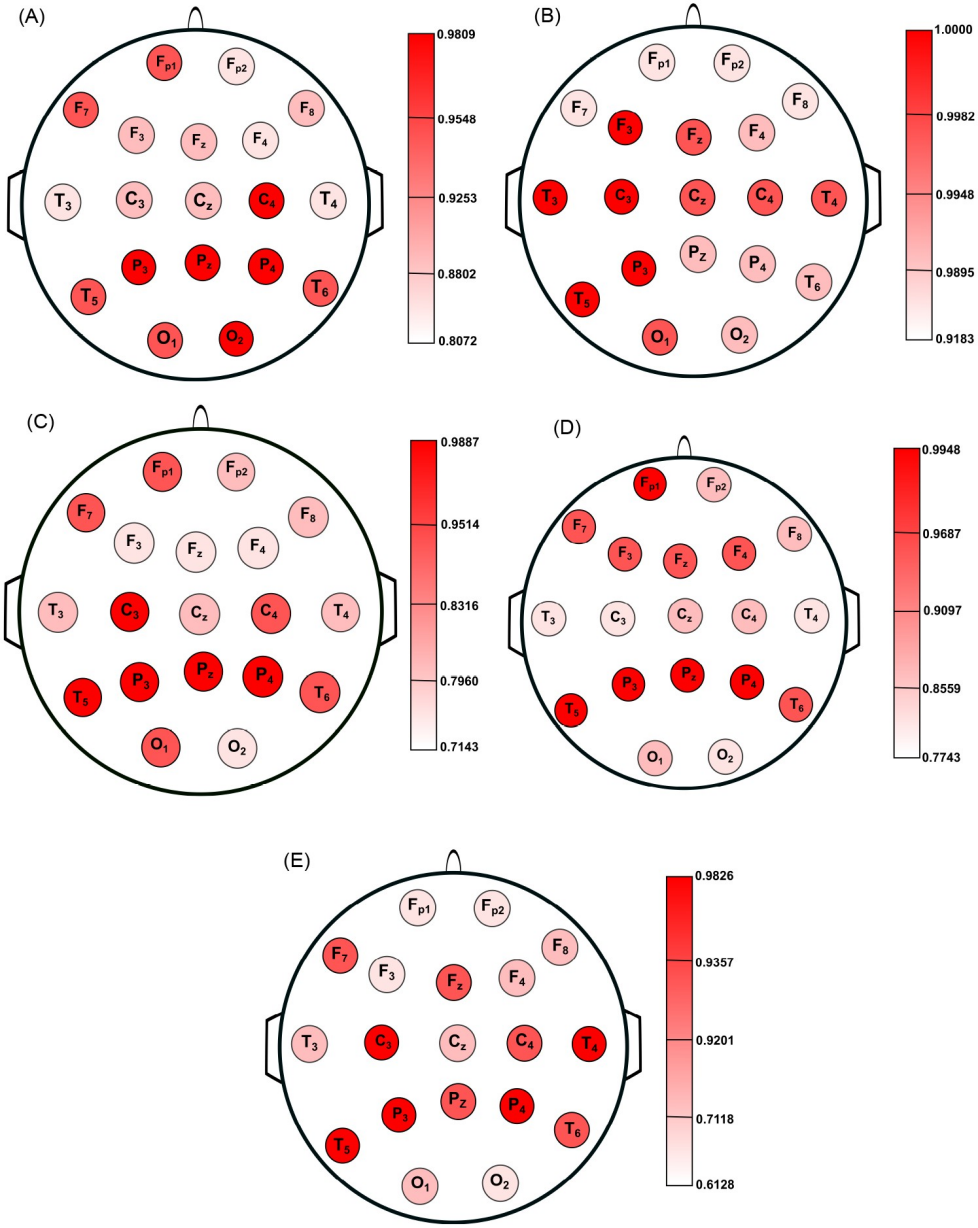
Location on scalp of the 19 EEG channels, represented by circles and colored according to the value of ${\hat{A}}_{ROC}$ for *CC* (A), Δ (B), *BC* (C), *Mo* (D), and *LEE* (E), respectively. Circles with darker colors indicate a better differentiation between aging and AD.

Fig 5 shows the location on scalp of the 19 EEG channels, colored according to the value of ${\hat{A}}_{ROC}$ for *CC*, Δ, *BC*, *Mo*, and *LEE*, respectively. The color map indicates values close to one for ${\hat{A}}_{ROC}$ in all cases, which means that the QG method was effective in differentiating normal controls from patients with AD, regardless of the network measure and the electrode location. Overall, Δ is the metric that displays the best results with $0.9183\leq {\hat{A}}_{ROC}\leq 1.000$. This result corroborates the knowledge that all patients under study display clear symptoms of the disease. Regardless of the network measure, the brain damage was found mostly in the parietal lobes and some loci in the temporal lobes (*T*_5_ and *T*_6_).

### 5.3 EEG wave patterns in the AD detection

It is widely accepted that Alzheimer’s disease earliest changes are an increase in theta activity and a decrease in beta activity, which are followed by a decrease in alpha activity [53, 54]. Delta activity increases later during the course of the disease [53]. In particular, the increase in theta activity is a typical finding in mild AD. The increase in delta activity is not evident until the more advanced stages of the disease take place [16, 54–56]. We apply the QG method to check if it can capture the influence of the EEG wave patterns in the AD development. Since the alpha rhythm increases in amplitude at rest with eyes closed [57], groups B and D were used in the analysis. Moreover, the data from channel *P*_3_ was chosen in the simulations due to its closeness to the parietal region, which is one of the regions to be later affected by AD.

Wavelet digital filter [58, 59] were used to extract the four EEG frequency bands, i.e., delta (1-4 Hz), theta (4-8 Hz), alpha (8-13 Hz), and beta (13-30 Hz). Analogous to previous cases, as all time series have the same length (*T* = 1, 024), we used *Q* = 2(1, 024)^1/3^ ≈ 20 and *k* = 1, 2, …, 25 in all calculations. Thus, we mapped 4 × 24 × 25 time series into 2,400 quantile graphs (or 2,400 *A*_*k*_ matrices), and therefore, we obtained 2,400 *W*_*k*_ matrices with *Q*^2^ = 400 elements each. Following, for each group and wave and for a given *k*, we computed the median over all weighed directed adjacency matrices *A*_*k*_ and obtained the Markov transition matrix of medians.

**Fig 6:**
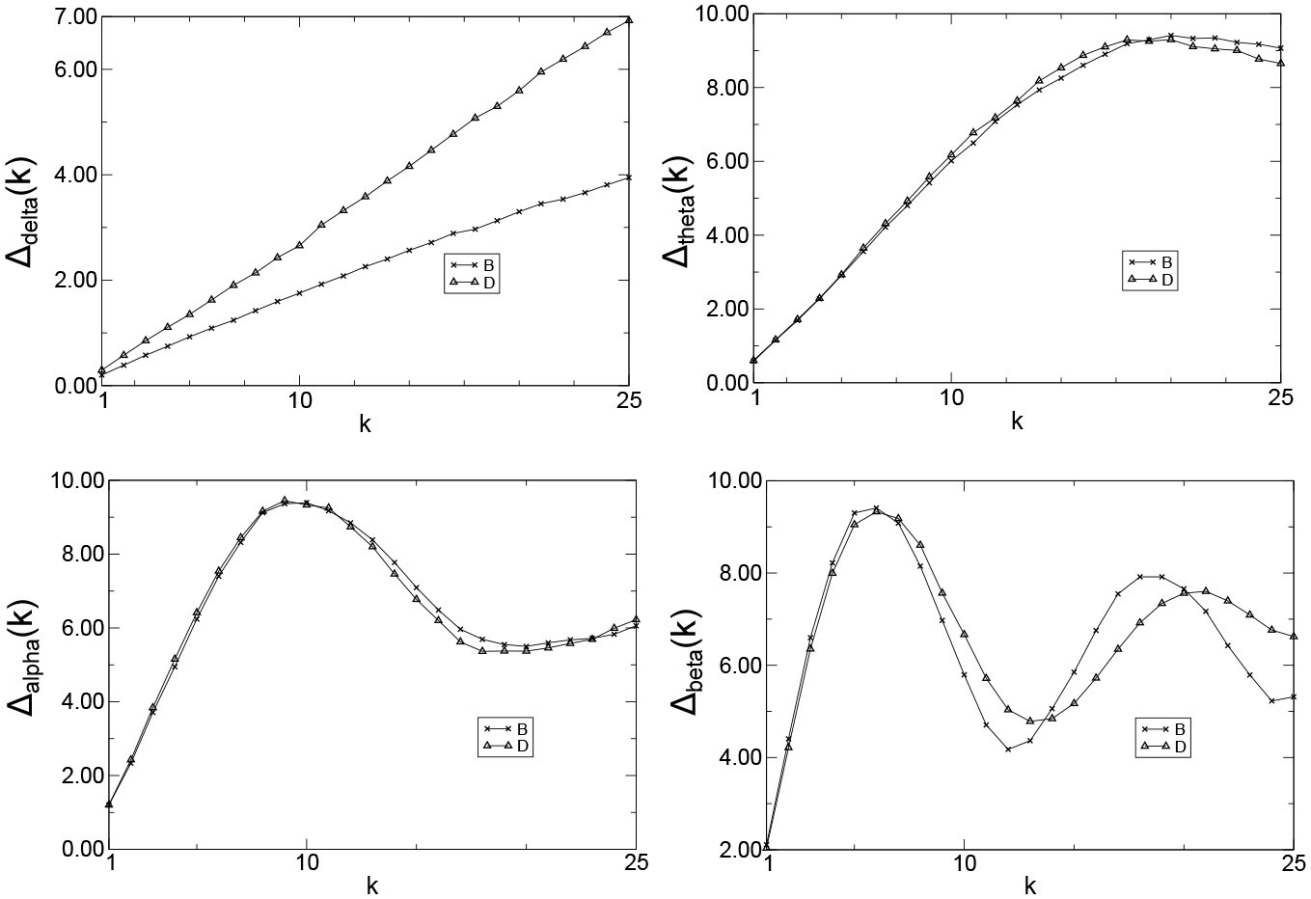
Δ(*k*) versus *k* (channel *P*_3_),*T* = 1, 024, *Q* = 20, and *k* = 1, 2, …, 25 for delta (Δ_*delta*_), theta (Δ_*theta*_), alpha (Δ_*alpha*_), and beta (Δ_*beta*_) waves and patients for the groups B and D.

For all groups and frequency bands, the mean jump length, which is the metric that best discriminates the groups in our analysis, was computed (Fig 6). Observe that the curves for normal controls (B) and patients with AD (D) are very similar for theta, alpha, and beta waves, regardless the value of *k*. On the other hand, there was statistically significant difference (95% confidence interval (CI) and a *p*-value of less than 0.05) between the sample means in groups B and D for delta waves. (Table 3). This result confirms the prior knowledge that all patients under study display clear symptoms of the disease and may have it in its late stage.

**Table 3:** Statistical comparison between the sample means of the network measure Δ for the groups B and D.

Waves	Delta	Theta	Alpha	Beta
*CI*_*BD*_	[0.4701; 2.3893]	[-1.6573; 1.7063]	[-1.2420; 1.1546]	[-0.8414; 1.1609]
*p* − *value*	0.0045	0.9767	0.9418	0.7497

## 6 Conclusion

Presently, there is no conclusive technique for the accurate diagnosis of AD [60, 61], a highly incapacitating disease. Thus, an automatic computer implemented technique based solely on the analysis of EEG data would potentially have a broad application. Building upon the work described in [33], in this study we presented an application of QG method to the analysis of EEG data. QGs map frequency, amplitude, and correlation characteristics of a time series (such as the EEG data of an AD patient) into the topological features of a network. The five network topological measures used here showed that the QG method is capable of discriminating health controls (with eyes open or closed) from patients with AD (with eyes open or closed), and indicate which channels (corresponding to 19 different locations on the patients’ scalp) provide the best discriminating power. All five network topological measures were able to generate statistically robust positive AD diagnostics, although the mean jump length provided the best results. Moreover, the combination of the network measures with a machine learning technique achieved outstanding performance in the two-class pattern classification problem presented here.

Spatially, the electrodes that best captured the symptoms were those nearer to the left and temporal-parietal chains. This observation is in line with the current understanding of the AD progression. Generally, AD mainly affects the left side of the temporal-hippocampal network, which is responsible for verbal memory and, apparently, is a more vulnerable hemisphere [62]. Furthermore, the joint analysis of delta, theta, alpha, and beta wave results indicate that all AD patients under study display clear symptoms of the disease and may have it in its late stage, a diagnosis known a priori and supported by our study.

In conclusion, we can say that the set of results presented in this paper attests that the QG method is an effective technique for the complex temporal pattern analysis like those found in EEGs from AD patients. It is worth mentioning that the subjects under study were not submitted to a definitive pathological diagnosis of AD as well as health controls. Some clinical features were not available at the time of this investigation. Moreover, the number of subjects is quite small limiting the extrapolations of the findings. Therefore, further research is necessary, with a larger and richer data set, to estimate the efficacy of the QG method in providing an early diagnostic of AD patients with only mild cognitive impairment.
